# Cancer Prevention and Therapy with Polyphenols: Sphingolipid-Mediated Mechanisms

**DOI:** 10.3390/nu10070940

**Published:** 2018-07-21

**Authors:** Michele Dei Cas, Riccardo Ghidoni

**Affiliations:** Department of Health Sciences, University of Milan, 20142 Milan, Italy; michele.deicas@unimi.it

**Keywords:** sphingolipids, ceramide, flavonoids, resveratrol, genistein, curcumin, nutrients, nutraceuticals, chemotherapeutics

## Abstract

Polyphenols, chemically characterized by a polyhydroxylated phenolic structure, are well known for their widespread pharmacological properties: anti-inflammatory, antibiotic, antiseptic, antitumor, antiallergic, cardioprotective and others. Their distribution in food products is also extensive especially in plant foods such as vegetables, cereals, legumes, fruits, nuts and certain beverages. The latest scientific literature outlines a resilient interconnection between cancer modulation and dietary polyphenols by sphingolipid-mediated mechanisms, usually correlated with a modification of their metabolism. We aim to extensively survey this relationship to show how it could be advantageous in cancer treatment or prevention by nutrients. From this analysis it emerges that a combination of classical chemotherapy with nutrients and especially with polyphenols dietary sources may improve efficacy and decreases negative side effects of the antineoplastic drug. In this multifaceted scenario, sphingolipids play a pivotal role as bioactive molecules, emerging as the mediators of cell proliferation in cancer and modulator of chemotherapeutics.

## 1. Polyphenols

### 1.1. Polyphenols: Chemical Classification

Polyphenols are one of the biggest class of phytochemicals (more than 8000 compounds) chemically characterized by common polyhydroxylated phenolic structures. Polyphenols are easily found in many plant-based products [1].

They can be divided into two main classes: flavonoids and non-flavonoids (Table 1). Flavonoids generally contain two phenolic rings (A and B rings) connected by a carbon chain or, more commonly, by an O-ring (C ring) which is similar to a phenylbenzopyrane structure. Based on the respective position of the B and C rings, functional groups and the presence of unsaturation in the C ring, they have been separated into subclasses: flavones, isoflavones, flavanones, flavonols, anthocyanidins, chalcones and flavanols, containing catechins and tannins. Hydroxylation and conjugation patterns characterize individual compounds in each subclass. In nature polyphenols (flavanols are an exception) exist as glycosides or other conjugates. Polyphenols can polymerize into large molecules, such as tannins, which are able to bind and precipitate proteins. The most important subclasses of tannins are proanthocyanidins, derived tannins and hydrolyzable tannins. Proanthocyanidins consist of monomeric units of flavans which are linked through carbon-carbon and ether linkages. They also may contain gallates. Relevant proanthocyanidins are procyanidins ([epi]catechin polymers), prodelephinidins ([epi]gallocatechin polymers) and propelargonidins ([epi]afzelechin). There is a second class of tannins which is comprised of tannins formed primarily beneath aerobic conditions during the manipulation of plants and subsequent processing into foods such as oolong and black teas, red wines and coffee. Important members of this subclass are theaflavins and thearubigins, easily found in tea. The last subclass of tannins comprises hydrolyzable tannins namely esters of gallic acid (gallotannins) or ellagic acid (ellagitannins) with a non-aromatic polyol [2].

**Figure 1:**
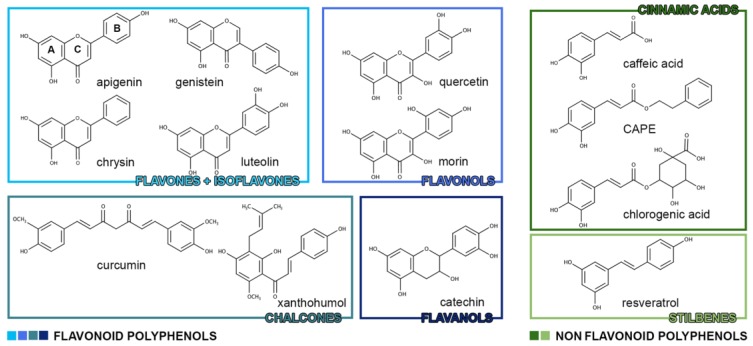
Chemical structures of polyphenols that are connected with a sphingolipid-based mechanism for cancer prevention and treatment.

Non-flavonoid polyphenols are divided into three main classes: phenolic acids (benzoic acid derivatives and cinnamic acid derivatives), stilbenoids and other polyphenols. Phenolic acids can be further divided, depending on the number of carbons, into two subclasses: benzoic acid derivatives (7 atoms of carbon) and cinnamic acid derivatives (9 atoms of carbon). In fruits and vegetables they are in a free-form whereas in grains and seeds they are in a conjugated form, that could be hydrolyzed by acid, alkaline or enzyme catalysis [1]. Stilbenoids class includes basic stilbenes, bibenzyls or dihydrostilbenes, bis(bibenzyls), phenanthrenes, 9,10-dihydrophenanthrenes and related compounds derived from the phenylpropanoid pathway. Stilbenes are structurally identified by a 1,2-diphenylethylene nucleus. They exist as both monomers and complex oligomers. The common monomeric skeleton consists of two aromatic rings linked by an ethylene bridge, commonly in *trans* configuration. The oligomers are formed by stilbene units (resveratrol-oxyresveratrol, resveratrol-piceatannol, resveratrol-isorhapontigenin, oxyresveratrol-isorhapontigenin and piceatannol-isorhapontigenin) linked by either C-C or C-O-C bonds [3]. Figure 1 shows the chemical structures of polyphenols considered in this review article.

### 1.2. Distribution of Polyphenols in Food

Many plants and herbs consumed by humans are known to contain relevant amounts of polyphenols, which have been demonstrated to have many beneficial effects such as anti-inflammatory, antibiotic, antiseptic, antitumor, antiallergic, cardioprotective and others. They are ubiquitous in plant foods such as vegetables, cereals, legumes, fruits, nuts and beverage such as wine, cider, beer, tea, cocoa. Their levels are mainly influenced by genetic factors, environmental conditions, variety, cultivars, processing and storage [4]. Specifically, the greatest dietary sources of flavonoids are tea (*Camellia sinensis*), onions (*Allium cepa*), apples (*Malus domestica*), citrus fruits (*Citrus* spp.), berries (blackberry *Rubus ulmifolius*, blueberry *Vaccinium* spp., elderberry *Sambucus* spp., raspberry *Rubus* spp., strawberry *Fragaria × ananassa*), legumes (*Fabaceae* spp.) and red wine (*Vitis vinifera*). Human flavonoid intake was estimated in the USA to be approximately 170 mg/day and in Netherlands 23 mg/day (both expressed as aglycones) using the content of only five flavonoids (quercetin, kaempferol, myricetin, luteolin and apigenin). Consequently, the effective intake may be much higher [5]. The dietary consumption of polyphenols consists principally of 80% flavanols, 8% for flavonols, 6% for flavanones, 5% for anthocyanidins, and less than 1% for isoflavones and flavones [6]. The major dietary sources of stilbenes are grapes and red wine (*Vitis vinifera*). Within this family resveratrol (Res) derivatives predominate, with several patterns of oligomerization and glycosylation [3]. For benzoic acid derivatives, the dietary sources were especially açaí oil (obtained from the fruit of *Euterpe oleracea*) [7], wine and vinegar [8]. For cinnamic acid compounds the food distribution was abundantly widespread: cereal grains, rice (*Oryza sativa*), wheat bran, coffee (*Coffea Arabica*), sweet potato (*Ipomoea batatas*), artichoke (*Cynara cardunculus*), cinnamon (*Cinnamomum cassia*), citrus fruits (*Citrus* spp.), grape (*Vitis vinifera*), tea (*Camellia sinensis*), cocoa (*Theobroma cacao*), spinach (*Spinacia oleracea*), celery (*Apium graveolens*), brassicas vegetables (*Brassicaceae* spp.), peanuts (*Arachis hypogaea*), basil (*Ocimum basilicum*) and garlic (*Allium sativum*) [9].

### 1.3. Bioavailability, Absorption and Metabolism of Polyphenols

The absorption and metabolism of polyphenols are consequent to: their chemical structure, the degree of glycosylation/acylation, the molecular size, the degree of polymerization and solubility [10]. Polyphenolic compounds can be distinguished into extractable and non-extractable according to their molecular weight and solubility: extractable polyphenols have a low-medium molecular mass and can be extracted using different solvents, whereas non-extractable remain insoluble due to their high molecular weight or complex phenols structures. Non-extractable polyphenols were highly recovered in feces, confirming the lack of absorption/digestion [11]. Concerning their metabolism, aglycones and simple monomeric polyphenols can be absorbed through the intestinal mucosa. On the other hand, glycosides cannot be absorbed because mammals lack in the proper β-glycosidases. However, some glycosides can be partially absorbed by the intervention of an enzyme present in the gastrointestinal microbiota [12]. Polyphenols undergo liver-mediated metabolism: methylation and/or conjugation with glucuronic acid or sulfate. Metabolites were secreted in the urine or in the bile, according to their lipophilic nature. In bile, some of them can be deconjugated and reabsorbed for many times (enterohepatic cycle) [13]. The level of absorbed polyphenols in the body and consequently their potential physiologic effects are still not clear [11,14].

## 2. Sphingolipids

### 2.1. Sphingolipid Classification

Sphingolipids are a complex family of amino alcohols compounds sharing a common structure: a sphingoid base backbone that is synthesized *de novo* from serine and acyl-CoA [15]. Sphingolipids can be divided into several different classes: sphingoid bases, ceramides, phosphosphingolipids, phosphonosphingolipids, neutral glycosphingolipids, acidic glycosphingolipids, including gangliosides, basic glycosphingolipids, amphoteric glycosphingolipids, arsenosphingolipids and others. The major sphingoid base of mammals is commonly referred to as sphingosine (Sph), that is (2*S*,3*R*,4*E*)-2-aminooctadec-4-ene-1,3-diol. Sphingoid bases found in nature could diverge in alkyl chain length and branching, the number and positions of unsaturation, the presence of additional hydroxyl groups and other features. These differences are mostly related to their specific role as for example by skin phytoceramides enriched in hydroxylation. Thus, interaction with nearby molecules strengthens the permeability barrier of the skin. In addition, a large number of fungi and sponges produces compounds with structural similarity to sphingoid bases some of which (such as myriocin and the fumonisins) are potent inhibitors of the enzymes of sphingolipid metabolism. In mammals sphingolipids are mainly represented by sphingomyelins (SM) which are formed by a polar head of phosphocholine and a core of ceramide (Cer). The latter is formed by a sphingosine amide-linked to fatty acids, mostly saturated or monounsaturated, bearing from 14 to 26 carbon atoms [16]. The major sphingolipid in insects is Cer-phosphoethanolamines whereas fungi have phytoCer-phosphoinositols and mannose-containing head groups [17]. SM is a dominant structural molecule, not only in plasma membrane but also in ER-to-Golgi vesicles as well as in membrane buddings (endocytosis and exocytosis). A high ratio of SM over cell lipids is present in blood cells, platelets and in the eye lens, which exhibits a peculiar increase of dihydro-sphingomyelin [18]. Cer is the central core of another important class named glycosphingolipids, in which Cer links one or more uncharged sugars such as glucose, galactose and fucose or modified sugars such as N-acetylglucosamine and N-acetylgalactosamine. Gangliosides are particular glycosphingolipids showing N-acetyl or N-glycolyl neuraminic acid as glycol-residues. Finally, there are basic glycosphingolipids and amphoteric glycosphingolipids. Water-living organism replace the phosphate polar group with either phosphono-group or arsenic acid. Sphingolipids can be linked to proteins, such as the inositol-phospho-Cers that are used by fungi to anchor membrane proteins and ω-hydroxyCers and ω-glucosylCers that are attached to surface proteins in human epidermal cells [17].

### 2.2. Sphingolipid Metabolism

Sphingolipids are synthesized in eukaryotic cells in the endoplasmic reticulum (ER) through a multiple step process whose rate is limited by serine palmitoyltransferase (SPT), an enzyme that catalyzes the initial condensation of serine with palmitic acid, forming 3-keto-sphinganine (3-KDS) [19]. Hereditary sensory and autonomic neuropathy type 1 has been recently associated with SPT mutations that enhance the affinity of the enzyme for alanine, instead of serine, thus forming neurotoxic deoxysphingolipids [20]. Reduction of 3-KDS by 3-KDS reductase (KDSR) releases dihydrosphingosine (or sphinganine, DHSph), which can be differentially acylated to form dihydroceramide (DHCer). Acylation is catalyzed by six different Cer synthases (CerS) [21] each using specific acyl chains, typically with saturated or mono-unsaturated fatty acids with 14 to 26 carbons. Cers are then formed by dehydrogenation *via* DHCer desaturase (DHCD). The enzymes involved in the Cer biosynthesis are included in the ER while in the Golgi occurs: (1) the synthesis of SM; (2) the synthesis of glycosphingolipids; and (3) the unusual phosphorylation of Cer, by Cer kinase (CerK), to Cer-1-phosphate (Cer-1P). Cer can be translocated from ER to Golgi by vesicular transport or anchored to a protein transporter (CERT). The transport *via* CERT was demonstrated to be specific for SM synthesis whereas vesicular trafficking for glycosphingolipids synthesis [22,23].

**Figure 2:**
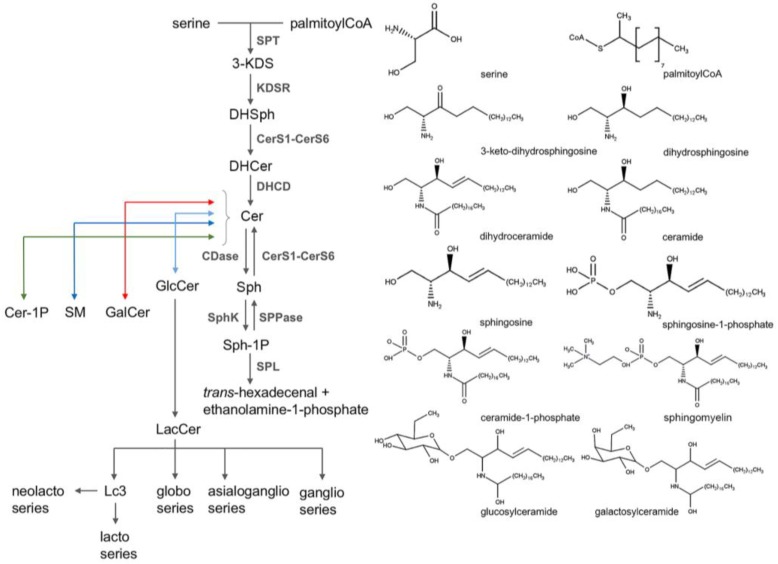
Sphingolipids metabolism and their chemical structures. Lc3: GlcNAcβ1-3Galβ1-4Glcβ-Cer for others see the abbreviation list.

SM is synthesized through the transfer of the phosphocholine head group of phosphatidylcholine to Cer by two enzymes: SM synthase 1 (SMS1) and 2 (SMS2). SMS1 is responsible for the *de novo* synthesis of SM whereas SMS2 probably resynthesizes SM from Cer generated by the catabolism of SM [22]. The whole production of glycosphingolipids starts from two direct derivatives of Cer, galactosylceramide (GalCer) and glucosylceramides (GlcCer). From the latter it is produced lactosylceramide (LacCer), that is the precursor of the neolacto-, lacto-, globo-, asialoganglio- and ganglio- series of glycosphingolipids [23]. Sphingolipids have a rapid turnover and their levels are constantly in change between synthesis and degradation. They are degraded in lysosomes by glycosidases or acid sphingomyelinases (aSMase) which remove the head groups to form Cers. Deacylation of Cer by ceramidases (CDase) is the only pathway known to generate Sph, that can be recycled back to Cer. Sph could also be phosphorylated by Sph kinases (SphK1 and SphK2) forming Sph-1-phosphate (Sph-1P). Sph-1P could either be dephosphorylated by phosphatases (SPPase1 and SPPase2) or degraded by Sph-1P lyase (SPL) to ethanolamine-1-phosphate and *trans*-hexadecenal. Sphingomyelinase (SMase) cleaves SM to Cer and phosphatidylcholine by a reversible reaction. Five types of SMase have been described and classified on their cation dependence and pH optima of action. The more relevant are Mg-dependent neutral sphingomyelinase (nSMase) and lysosomal aSMase [24]. An overview of sphingolipids metabolism and chemical structure of the principal ones is shown in Figure 2.

### 2.3. Sphingolipids Modulation of Cellular Functions

The structural diversity of sphingolipids reflects a correspondent diversification in pathophysiological functions: regulation of apoptosis [25], proliferation, differentiation, autophagy [26], invasiveness, modification of signaling cascade and mediation of inflammatory responses by cytokines [27,28].

Cer promotes cell-type specific apoptosis by (1) activating both protein kinases such as protein kinase C (PKC), protein phosphatases 1-2 and proteases, including caspases and cathepsin D; (2) formation of pores in the mitochondrial membrane; and (3) modulation of pro-apoptotic Bcl-2-family proteins [29,30]. Also, Sph *via* PKC upholds apoptosis [31].

In contrast to Cer, that is predominantly pro-apoptotic, Sph-1P is mainly an anti-apoptotic messenger by stimulating G-protein-coupled receptors activating RAS, RAC, phosphatidylinositide 3-kinases (PI3K), protein kinase B (AKT), phospholipase C (PLC) and Rho kinase. The regulation of a signaling cascade mediated by Sph-1P includes modulation in mitogenesis, cell migration, cytoskeletal rearrangement and angiogenesis. Sphingolipids could also be correlated with pro-inflammatory cytokines through different mechanisms [31,32]. Sph-1P stimulate inflammation by either upregulation of cyclooxygenase 2 (COX-2) with overproduction of prostaglandin E_2_ and activation of nuclear factor kappa-light-chain-enhancer of activated B cells (NF-κB). In the same way, Cer-1P through activation of cytosolic phospholipases A2 (cPLA2) enhances the production of pro-inflammatory arachidonic acid [33].

### 2.4. Sphingolipids and Cancer

Sphingolipids have emerged as mediators of cell proliferation in cancer and as potential chemotherapeutics (Table 2). In general, Cer regulates anti-cancer cellular fate whereas Sph-1P is pro-oncogenic and pro-metastatic.

Cer normally mediates antiproliferative responses such as cell growth inhibition, apoptosis induction, senescence modulation, ER stress response and autophagy. Interestingly, recent studies [34,35] suggest that *de novo*-generated Cers present an ambivalent role in the promotion/suppression of tumors reliant to their fatty acid chain lengths, subcellular localization and direct downstream targets. In a study [36] on head and neck squamous cell carcinoma (HNSCC) decreased levels of C18 Cer are correlated with lymphovascular invasion and nodal metastasis. Conversely, overexpression of CerS1 and increased levels of *de novo* synthesized C16 Cer show a reduction of tumoral cell growth by inhibition of telomerase activity. Overexpression of *de novo* synthesized C16 Cer was associated with tumor proliferation whereas downregulation of *de novo* synthesized C16 Cer induce ER stress and apoptosis of HSNCC cells by activating the ATF6/CHOP pathway. Furthermore, elevated levels of C16 Cer, CerS2 and CerS6 were associated with breast cancer. Moreover, the interaction of Cer with cathepsin D, PKC, I2PP2A, caspases and telomerase leads to apoptosis, growth suppression and senescence.

Cer-1P has been shown to induce the release of arachidonic acid in cancer cells leading to an inflammatory condition [37].

SM contributes to release diacylglycerol from phosphatidylcholine, a well-known activator of PKC, thus promoting cellular proliferation. GlcCer indeed leads to drug resistance.

Sph-1P induces anti-apoptosis processes engaging with Sph-1P receptors 1–5 (S1PR1–5). In addition, elevated levels of Sph-1P have been observed in different cancer and tumor tissues [38,39].

The SphK1 expression has been found to be upregulated in a number of solid tumors. High levels of SphK1 has been correlated with poor survival of patients who suffer from glioblastoma, gastric and breast cancers. In accordance, anticancer regimens have been shown to down-regulate SphK1 activity in various cancer cell and animal models. This enzyme-increased transcription is proposed to be responsible for chemo- and radio-resistance of cancer cells and to favor the progression of hormone-refractory state. As an example, it was proved a direct correlation of SphK1 activity and expression with prostate tumor grade as well as with the clinical outcome after prostatectomy [40].

## 3. Focus on Cancer: Dietary Polyphenols and Sphingolipids

### 3.1. Apigenin

Apigenin (4′,5,7-trihydroxyflavone) is a flavone found in fruits, vegetables and other plants. It counteracts inflammation, oxidative stress and development of cancer [41]. Major apigenin-containing food sources include thyme (*Thymus vulgaris*), cherries (*Prunus avium*), tea (*Camellia sinensis*), olives (*Olea europaea*), broccoli (*Brassica oleracea*), celery (*Apium graveolens*), and legumes (*Fabaceae* spp.). The most abundant sources are the leafy herb parsley (*Petroselinum cripspum*) and dried flowers of chamomile (*Matricaria chamomilla*) [42]. Although a few contradictory reports [43,44], apigenin exerts anti-tumoral effect influencing mitochondria activity, gene expression and partially through targeting of the JAK/STAT pathway [45].

Moussavi et al. [46] investigated the effect of apigenin as a dietary component in colon cancer by testing its relationship with cell death, mediated alternately by Cer and reactive oxygen species (ROS).

**Figure 3:**
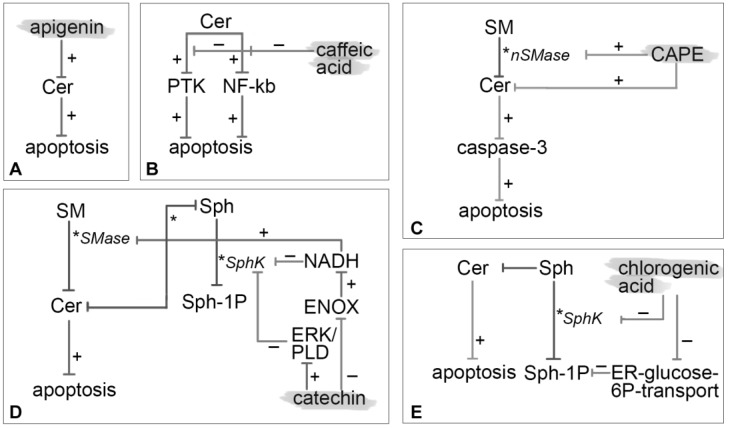
Mechanism of modulation on sphingolipids by apigenin (**A**), caffeic acid (**B**), CAPE (**C**), catechin (**D**) and chlorogenic acid (**E**). It is depicted with an asterisk (*) enzymatic pathway, with plus (+) red-regulated pathway and with minus (−) down-regulation ones. PTK: protein tyrosine kinase.

Apigenin was reported to elevate Cer levels and apoptosis in colon cancer cells (HCT116) in a concentration- and time-dependent manner but independently on the *de novo* synthesis pathway (Figure 3A).

### 3.2. Caffeic Acid

Caffeic acid (3,4-dihydroxycinnamic acid) is a widespread hydroxycinnamic acid, naturally found in many plant species as a secondary metabolite of the shikimate pathway. It displays the classical framework of phenylpropanoids (C6-C3) with a 3,4-dihydroxylated aromatic ring connected to a carboxylic acid moiety by a *trans*-ethylene ether. It is the most abundant hydroxycinnamic acid and the diet sources are argan oil (*Argania spinosa*), oats (*Avena nuda*), wheat (*Triticum* spp.), rice (*Oryza sativa*), olive oil (*Olea europaea*), narrow-leaved purple coneflower (*Echinacea angustifolia*), and berries (blackberry *Rubus ulmifolius*, blueberry *Vaccinium* spp., elderberry *Sambucus* spp., raspberry *Rubus* spp., strawberry *Fragaria × ananassa*). Other dietary sources include potatoes (*Solanum tuberosum*), carrots (*Daucus carota*), artichokes (*Cynara cardunculus*) and obviously coffee (*Coffea arabica*) [47,48]. The average phenolic acids intake in humans is in the order of 210 mg/day within a broad range, depending on nutritional habits. Caffeic acid has been reported to account for up to 90% of total phenolic acids intake [49]. The wide spectrum of biological effects induced by caffeic acid includes: enzyme activity inhibition (5- and 12-lipoxygenases, glutathione S-transferase, xanthine oxidase), antitumor activity, anti-inflammatory properties, modulation of cellular response to ROS and inhibition of HIV replication [50,51,52].

Nardini et al. [50] reported that caffeic acid significantly inhibits Cer-induced activation of NF-κB in human monocytic U937 cells, with consequent suppression of acute inflammation, septic shock, HIV replication, acute phase response, viral replication, radiation damage, atherogenesis and possibly some neoplastic degeneration. The NF-κB inhibition mechanisms may be different: countering the changes of the intracellular redox status induced by Cer, inhibition of 5 and 12 lipoxygenases activities or PKC and PKA activity arrest. Additionally, some data indicate that caffeic acid inhibits protein tyrosine kinase activity [53,54]. This ability may be the mechanism liable for the inhibition of Cer-induced apoptotic response rather than its antioxidant properties. This hypothesis was also in agreement with the observation that no tested antioxidants inhibit DNA fragmentation and therefore apoptosis. The action of caffeic acid is two-faced: it shows pro-apoptotic effects at high concentrations (>200 μM) and antiapoptotic ones at lower levels explaining a conflicted range of activities [50]. At low concentrations, close to those expected *in vivo*, it mediates a double inhibition mechanism on Cer-induced NF-κB activation and Cer-induced apoptosis by protein tyrosine kinase. Under this perspective, caffeic acid could not be used as a coadjuvant to chemotherapy in low concentrations since it reduces Cer-mediated apoptosis (Figure 3B).

### 3.3. CAPE

Caffeic acid phenethyl ester (CAPE) or 2-phenylethyl (2E)-3-(3,4-dihydroxyphenyl)acrylate is a natural bioactive compound. It occurs in many plants and the main human source is propolis. Propolis is a resinous substance made by honeybees mixing saliva, beeswax and exudate collected from botanical sources. CAPE is a cinnamic acid polyphenol characterized by a hydroxyl catechol ring. It has different biological activities on infections, oxidative stress, inflammation, cancer, diabetes, neurodegeneration and anxiety [55].

Tseng et al. [56] demonstrated that CAPE-induced apoptosis involves nSMase activation and accumulation of Cer in C6 glioma cells. CAPE modulates two parallel signaling pathways both leading to activation of caspase 3 as an ultimate effector of apoptosis. On one hand CAPE increases nSMase activity triggering the activation of ERK/NGFR/NGF/JNK pathway and on the other hand it causes an accumulation of Cer which initiates the p38 MAPK/p53/BAX signaling path. In addition to the apoptotic potential of CAPE in cancer cells a coherent manipulation of Cer levels may improve the efficacy of chemotherapy agents (Figure 3C). 

### 3.4. Catechin

The catechin family presents two benzene rings and a 3-OH-dihydropyran heterocycle with two chiral centers on C2 and C3. Thus, it has four diastereoisomers: two in *trans* configuration called catechin and two in *cis* configuration called epicatechin. In plants they are usually conjugated with gallic acid.

Epigallocatechin-3-gallate (EGCG) is the most potent catechin with antioxidant properties and it is mainly present in green tea together with its related compounds epicatechin [57]. High concentrations of catechin can be found in fresh tea leaves (*Camellia sinensis*), red wine, broad beans (*Vicia faba*), black grapes, apricots (*Prunus armeniaca*) and strawberries (*Fragaria × ananassa*) nevertheless epicatechin could be found in high concentrations in apples (*Malus domestica*), blackberries, broad beans (*Vicia faba*), cherries (*Prunus cerasus*), black grapes, pears (*Pyrus* spp.), raspberries (*Rubus* spp.), and chocolate (*Theobroma cacao*). Catechins showed *in vitro* protection against degenerative diseases and a strong inverse relationship between the intake of catechins and risk of mortality by cardiovascular heart diseases [58]. It has been reported that catechins have antimicrobic activity (gram-positive more than gram-negative) and inhibit carcinogenesis of the skin, lung, esophagus, stomach, liver, small intestine, colon, bladder, prostate, and mammary glands. EGCG has been described to have many potential targets for action against carcinogens and among them also sphingolipids [58].

Brizuela et al. [40] reported, for the first time, that green tea polyphenols (EGCG and polyphenon E, PPE) inhibit SphK1 activity, *via* a novel ERK/PLD-dependent mechanism in prostate cancer cells (C4-2B hormone-responsive and PC-3 hormone-refractory). The treatment with ECGC and PPE in both PC-3 and C4-2B cell lineages showed a remarkable inhibition of cell growth by altering the sphingolipid balance correlated with SphK1 inhibition and increment of pro-apoptotic Cer. The mechanisms underlying SphK1 inhibition by green tea extract are dependent on the down-regulation of the ERK1/2 and consequently with PLD/PA signaling pathway [40,59]. *In vivo* studies, confirmed the data obtained *in vitro*, suggesting that animals with SphK1 overexpressing PC-3 cells implanted in a subcutaneous district develop larger tumors and resistance to green tea due to disruption of sphingolipid equilibrium. In conjunction, EGCG and PPE diet is also associated with a significant metastasis reduction in the orthotopic PC-3 model. Preventive approaches [60,61] using catechins have been shown to inhibit other cancers as the colon one. Hence, a combination of green tea polyphenols and chemotherapeutic agents or radiation therapy would be promising.

Another mechanism of Cer-mediated apoptosis proposed by Wu et al. [62] involves ENOX2 (tNOX) inhibition by EGCG. Inhibition of the ENOX family commonly results in an accumulation of cytosolic NADH at the inner leaflet of the plasma membrane. Regarding sphingolipid metabolism, NADH modulates SphK inhibition and SMase stimulation. The disruption of sphingolipid rheostat, which is clearly connected with apoptosis, occurs when Sph-1P levels increase and Cer levels decrease (Figure 3D). 

### 3.5. Chlorogenic Acid

Chlorogenic acid, a non-flavonoid polyphenol, is a quinic acid conjugate of caffeic acid found in high levels in coffee beans (*Coffea arabica*). An average coffee drinker tends to consume 0.5–1 g of chlorogenic acids daily. It could be found also in apples (*Malus domestica*), pears (*Pyrus* spp.), eggplants (*Solanum melongena*), tomatoes (*Solanum lycopersicum*), blueberries (*Vaccinium myrtillus*), strawberries (*Fragaria × ananassa*), bamboo (Bambuseae spp.) and potatoes (*Solanum tuberosum*) [63,64]. It has various biological activities such as anti-inflammatory, anti-diabetic, anti-tumorigenic, antioxidative, anti-gout and anti-obesity.

Lee et al. [65] demonstrated that the inhibition of Hypoxia-Inducible factor-1α (HIF-1α) by chlorogenic acid involves the SphK-1 pathway under hypoxia in the DU145 human prostate cancer cell line. Hypoxia is a common condition in solid tumors enhancing its rough development. HIF-1α is a transcription factor that regulates cancer progression such as angiogenesis, metastasis, anti-apoptosis, cell proliferations whereby it imparts resistance to chemotherapy. SphK-1 regulates and stabilizes HIF-1α through the AKT/GSK-3 leading to his accumulation. It was shown that under hypoxia, chlorogenic acid significantly decreases HIF-1α and SphK-1 activity. Besides, it prevents phosphorylation of AKT and GSK-3β which are involved in stabilization of HIF-1α by SphK. In summary, chlorogenic acid decreased cancer cell growth by (1) inhibition of SphK-1 and reduction of HIF-1α; (2) decrement of phosphorylation of HIF-1α stabilizing agent; (3) decrease of VEGF (vascular endothelial growth factor) and angiogenesis.

Additionally, according to Belkaid et al. [66], chlorogenic acid possesses anticancer properties in highly invasive U-87 glioblastoma cells. The competitive inhibition of ER-glucose-6-phosphate transport was shown causing a consequent downregulation of Sph-1P-induced cell migration and a hindrance to Sph-1P-induced ERK phosphorylation. Sph-1P is present at high levels in brain tissue acting as a potent mitogen for glioblastoma multiform cells, triggering intracellular signaling by MAPK pathway and causing the release of intracellular calcium pools (Figure 3E).

### 3.6. Chrysin

Chrysin is a naturally occurring flavone found in human diet products such as *Passiflora caerulea*, *Passiflora incarnate* infuse, *Oroxylum indicum* and mushroom, *Pleurotus ostreatus* [67]. Traditional Chinese medicine uses the seed of *Oroxylum indicum* in the treatment of cough, acute or chronic bronchitis, pharyngitis, pertussis and other respiratory disorders. Other parts of this small tree such as leaves, flowers and immature boiled fruits are commonly used in the daily diet in Thailand and Laos. Baicalein, oroxylin A and chrysin which can be isolated from its bark play an important role in cancer, as well as viral and bacterial infections [68]. Chrysin has been shown to have a broad range of pharmacological effects such as anti-oxidation, anti-viral, anti-inflammatory properties and anti-cancer properties on breast cancer cells.

**Figure 4:**
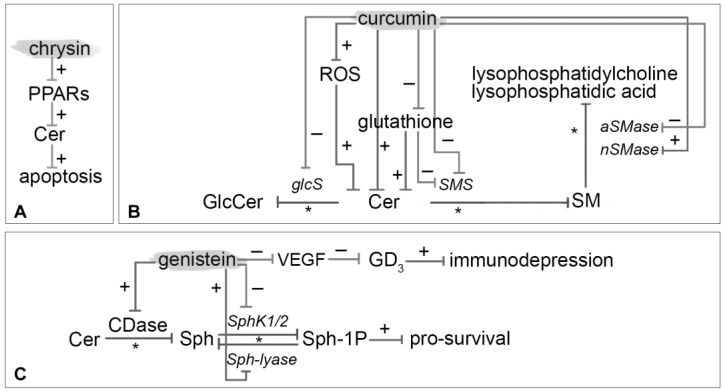
Mechanism of modulation on sphingolipids by chrysin (**A**), curcumin (**B**) and genistein (**C**). It is depicted with an asterisk (*) enzymatic pathway, with plus (+) red-regulated pathway and with minus (−) down-regulation ones.

Hong et al. [69] evaluated the effects of chrysin treatment on human estrogen receptor (ER)-negative breast cancer cells (MDA-MB-231). This study provides mechanistic evidence that chrysin treatment inhibits the cancer cell growth with a direct or indirect increased expression of PPARα mRNA. PPARs activation can result in intracellular accumulation of Cer, which mediates downstream effects such as apoptosis. Besides, Cer accumulation is assumed to be dependent on a modulation of arachidonic acid (Figure 4A) [70].

### 3.7. Curcumin

Curcumin is one of the main substances found in the rhizome of turmeric (*Curcuma longa*) and other *Curcuma* spp. Commercially available curcumin contains about 77% in curcuminoids that include pure curcumin, demethoxycurcumin and bis-demethoxycurcumin. [71].

Curcumin inhibits cell proliferation and stimulates apoptosis by affecting various key targets in signal transduction pathways, including Akt, cyclooxygenase, NF-kB, c-myc, Bcl-2, c-Jun N-terminal kinase (JNK), and epithelial growth factor (EGF) receptor (Figure 4B).

Cheng et al. [72] demonstrated that curcumin inhibits cell growth and induces apoptosis in colon cancer cells (Caco-2 cells) affecting aSMase activity. It reduces the hydrolytic capacity of the enzyme associated with a slight increase of cellular SM. No modification of alkaline, nSMase and phospholipase D was found after curcumin treatment. Reduction of aSMase activity was not due to a direct inhibitory effect of curcumin on the enzyme, but rather to an inhibition of the enzyme biosynthesis. The up-mentioned action is particularly evident in specific cell type: stronger in monolayer Caco-2 cells than in polarised ones. The role of aSMase in cancer is still debated and there is evidence suggesting that this enzyme activity may affect phospholipase A_2_ and thus the formation of lysophosphatidylcholine and lysophosphatidic acid which are required for colon cancer metastasis [73,74].

In contrast, Moussavi et al. [75] found that curcumin significantly increased the Cer levels in colon cancer HCT 116 cells without detectable changes of aSMase and nSMase. Cer generation by curcumin occurred through *de novo* synthesis since cell death could be reversed by myriocin, an inhibitor of serine palmitoyltransferase. Colon cancer cell apoptosis by curcumin was strongly related with JNK activation mediated principally by ROS generation and to a minor extent *via* a parallel Cer-associated pathway.

Another study on anti-colorectal cancer effects by curcumin was conducted by Chen et al. [76]. They showed that co-administration of curcumin and perifosine, an orally bioactive alkylphospholipid, increases colorectal cancer cell apoptosis by modulating multiple signaling pathways such as inactivation of Akt and NF-κβ, activation of c-Jun, downregulation of Bcl-2 and cyclin D1 and increment in intracellular levels of both ROS and Cer. Furthermore, they suggested that ROS/Cer production after co-administration of curcumin and perifosine and ER stress response were independent of Akt inhibition and Bcl-2/cyclin D1 downregulation.

Yu et al. [77] showed that curcumin-induced cell growth inhibition and apoptosis in melanoma cell lines (WM-115 and B16) could be facilitated by PDMP (DL-threo-1-phenyl-2- decanoylamino-3-morpholino-1-propanol). PDMP is a well-known inhibitor of sphingolipid biosynthesis especially directed to the formation of GlcCer, thus resulting in an accumulation of its endogenous precursor. Combination of PDMP and curcumin may be used as a new therapeutic intervention against melanoma. Curcumin induces an early increase of Cer (12 h), that melanoma cells could remove, after long-term (24 h), by glycosylation. Upon incubation on PDMP, Cer levels remain elevated causing further cell death and apoptosis. In addition, exogenous cell-permeable C6-Cer sensitizes melanoma cell lines to curcumin-induced apoptosis.

The curcumin effect was investigated in clinical trials of patients with multiforme glioblastoma, ideally as a second line therapy after failure of radiation and temozolomide [78]. The optimal method should be setting curcumin in combination with an established cytotoxic chemotherapy agent such as carmustine or lomustine. A progression of this aggressive brain cancer is related to a decrease in Cer levels: curcumin has been shown to enhance Cer production influencing CerS activity.

According to Thayyullathil et al. [79], curcumin has been shown to be a pro-autophagic drug in malignant gliomas. Malignant glioma cells are likely responding to therapy better *via* autophagy than apoptosis but, for apoptosis-resistant glioblastoma patients, a pro-autophagic drug could be extremely advantageous. Curcumin induces autophagy by Par-4 (prostate apoptosis response-4) upregulation and Cer generation *via* ROS-dependent mechanism. Cer generation was correlated to the nSMase pathway in U87MG malignant glioma cells since GW4869, an inhibitor of nSMase, significantly blocked curcumin-induced Cer generation and autophagy. 

Hilchie et al. [80] determined the mechanism by which curcumin induces cytotoxicity in prostate cancer cells (PC3). This treatment caused time- and dose-dependent apoptosis and depletion of cellular reduced glutathione, Cer accumulation, activation of p38, JNK and release of different caspases and cytochrome c. The authors conclude that apoptosis in prostate cancer is due principally to Cer accumulation causing mitochondrial membrane integrity damage, a consequent release of cytochrome c and apoptosis-inducing factor. By contrast, clinical trials have confirmed that curcumin is poorly absorbed in the gastrointestinal tract owing to the efficient efflux of monoglutathionyl curcumin conjugates from intestinal epithelial cells into the lumen. Achieving a useful plasma concentration to trigger apoptosis is the major obstacle to the clinical application of curcumin-based therapy. Combination of curcumin and piperine or more stable analogs of curcumin may overcome these pharmacokinetics problems.

Kizhakkayil et al. [81] investigated more deeply the glutathione decline as a mechanism by which curcumin acts on human leukemic cells. A decrease of intracellular glutathione regulates caspase-dependent inhibition of SMS activity and Cer generation, and thus apoptosis. Curcumin-induced Cer generation and apoptosis were inhibited by extracellular supplementation of glutathione, N-acetylcysteine and caspase inhibitor z-VAD-fmk, supporting these findings. In particular, an important role in Cer generation was found to be related to the regulation of the SMS cycle and not to the *de novo* pathway.

Scharstuhl et al. [82] revealed that curcumin induces apoptosis by the formation of channels in the outer mitochondrial membranes and the release of apoptosis-inducing factors. The formation of channels was correlated to the combined action of Cers, VDAC and BAX and not to caspases pathways. Nevertheless, inhibition of the *de novo* synthesis and inhibition of SMase did not significantly block curcumin-induced apoptosis, indicating that Cers are partially involved.

Shakor et al. [83] examined curcumin-induced apoptosis in human leukemia HL60 cells and their HL60/VCR multidrug-resistant counterparts. The molecular mechanism of curcumin action consists in a biphasic Cer accumulation in the cells firstly by rapid activation of nSMase2 and then by inhibition of SMS, accompanied in the drug-resistant cells by glucosylceramide synthase (GlcS, the enzyme involved in GlcCer synthesis from Cer) inhibition. The intracellular increase of Cer modulates the transcription of apoptosis-regulating genes, such as BAX, Bcl-2 and caspase-3. The glycosylation of Cer, *via* GlcS, is recognized as a chemoresistance strategy and enhanced by several tumors. On the other side, the down-regulation of this Golgi enzyme seems to be related to P-gp inhibition. P-gp, an ATP consuming flippase, translocates GlcCer. P-gp antagonists (cyclosporine A or tamoxifen) impair Cer clearance and enhance its cytotoxicity. Moreover, molecular modeling studies confirmed that curcumin binds to P-gp in its substrate binding site possibly competing with GlcCer binding. Finally, apoptosis is associated with Cer increase, glutathione depletion and ROS generation after curcumin treatments.

Another study by Shakor et al. [84] indicated a complex crosstalk among Bcl-2, Bcl-xL, caspases and glutathione during curcumin-induced apoptosis. This point to the superior role of caspase-8 activity, Bcl-xL down-regulation and glutathione depletion in the pro-apoptotic cascade leading to nSMase activation and hence generation of Cer. The signaling cascade controlling Cer-mediated apoptosis in curcumin-treated cells was: caspase-8 activation, Bcl-xL degradation, glutathione depletion, nSMase activation and Cer accumulation. Caspase-3 activation and Bcl-2 degradation, both regulated by glutathione levels and reciprocally interconnected, are also co-involved in SMase initiation. SMS degradation was indeed regulated only by caspase-3 activation. 

Yang et al. [85] analyzed the impact of the SphK1 inhibitor on Cer production, particularly as a potential curcumin chemo-sensitizer in ovarian cancer cells (CaOV3). Inhibition of SphK1, by pharmacological tools as SKI-II (2-(p-Hydroxyanilino)-4-(p-chlorophenyl)thiazole) or by RNA interference, dramatically enhanced curcumin-induced apoptosis and growth inhibition in ovarian cancer cells *via* Cer production and p38 activation and Akt inhibition.

A further supplement to curcumin treatment (Qui et al. [86]) was the addition of exogenous cell-permeable short-chain, C6-Cer. It sensitizes melanoma cells (B16 and WM-115) to curcumin-mediated apoptosis due to the augment of the mitochondrial apoptosis pathway, especially through (1) the cleavage of caspases 3 and 9 and (2) the downregulation of anti-apoptosis protein Bcl-xL and X-IAP.

### 3.8. Genistein

Genistein is essentially present in soy-derived products and the soybeans contain the compound in ranges from 5.6 to 276 mg/100 g. In addition to genistein soy foods contain another major isoflavone, daidzein. Daidzein differs from genistein by the lack of the hydroxyl group on position 5. Both isoflavones may exist in their aglycone or glycoside forms. The most common glycoside forms of genistein and daidzein are O-β-d-glucoside derivatives. Due to soy consume the average dietary isoflavone intake in Asian countries is in the range of 25–50 mg/day, whereas in Western countries, the intake is approximately 2 mg/day. In lower concentrations genistein and daidzein are also present in legumes. The genus *Lupinus* (commonly known as lupin) represents a typical example of the legume that is now widely cultivated for its seeds, which possess a nutritional value similar to soybean. Other important legumes are broad beans (*Vicia faba*) and chickpeas (*Cicer arietinum*), but the flavones can be detected also in fruit, nuts, and vegetables where their content can vary considerably, ranging being from 0.03 to 0.2 mg/100 g [87].

This soybean isoflavone exerts many cellular effects, namely apoptosis activation, and protein-tyrosine kinase activity and angiogenesis inhibition (Figure 4C). It is important to note that genistein affects in a dose-dependent manner, both positively and negatively tumorigenesis.

Engel et al. [88] reported the influence of phytoestrogens, such as genistein, on the metabolome of breast cancer cells. They compare either MCF-7, positive for ERα and ERβ, and MCF-12A, a non-tumorigenic epithelial breast cell line. Three sphingolipids were analyzed: Sph, DHSph and ethanolamine-phosphate. These metabolites were elevated in MCF-7 under control conditions and genistein treatment normalizes their levels. Whereas their amounts, in MCF-12A, were not affected. By contrast, DHSph was not normalized by genistein treatment in MCF-7 to gain the level of MCF-12A under control conditions. Western blotting-coupled immunofluorescence experiments revealed a significant, concentration-dependent, decrease in the amount of SphK1 and SphK2 enzyme in MCF-7 after genistein exposure. In MCF-12A phytoestrogen exposure revealed boosted SphK1 amounts and undetectable expression of SphK2. These findings suggested that SphK1 is expressed in cancerous as well as non-tumorigenic cells while Sphk2 is overexpressed in cancer line. SPL expression was also investigated. MCF-7 has a weaker expression than MCF-12A but after exposure with genistein, the SPL amount increases dramatically. Exposure to phytoestrogens in higher concentrations (10 μM of genistein) resulted in (1) decreased tumor progression *via* sphingolipids pathway and (2) enhanced the reaction of SPL causing a higher conversion of Sph-1P to phosphoethanolamine.

Lucki et al. [89] showed that nanomolar concentrations of genistein induces aCDase transcription in MCF-7 breast cancer cells *via* ERK1/2 dependent mechanism.

The proliferative properties of genistein are supposed to be related to its ability to stimulate estrogenic pathways by binding ERα and GPR30. GPR30 is a transmembrane G-protein-coupled receptor that binds most ER ligands triggering estrogenic signaling and proliferation. aCDase is a lipid hydrolase, that degrades Cer to Sph and a free fatty acid, thus playing a key role in cellular homeostasis regulation by controlling the Cer/Sph/Sph-1P balance within the cell. Activation of this pathway promotes: (1) histone acetylation; (2) recruitment of the phospho-estrogen receptor α; and (3) translocation of Sp1 transcription factor to the aCDase promoter. This activation culminated in an increased enzymatic activity, which results in increased Sph-1P production. Nanomolar concentrations of genistein stimulates the growth of ER-positive breast cancer cells by modulating expression of aCDase. Such modulation produces two synergic but different events: (1) an increment of Sph-1P levels, which activates proliferative pathways by binding to cell surface receptors and (2) the modulation of cyclin B2 expression, driving mitotic progression and cell growth.

Another study by Engel et al. [90] showed that high doses of genistein promote the growth of bone cancer cells. They explored the co-administration of genistein and calcitriol in order to inhibit immature osteosarcoma cells MG-63. The malignant proliferation induced by 100 μM genistein could be normalized to control levels after simultaneous exposure to 10 nM calcitriol. This synergistic effect may be consistent with (1) an overexpression of ERβ, (2) a reduction of extracellular acidification and respiration rates and (3) an increased ethanolamine production by the overexpression of SPL.

The use of genistein as an anti-cancer compound is usually limited because a relatively high concentration is necessary. Ji et al. [91] counteracted this limitation by adding exogenous cell-permeable short-chain Cers to enhance genistein activity. In this study, melanoma cell line (B16, WM451, MeWo) were sensitized to genistein by increasing cellular level of Cers, both exogenously and endogenously. In B16 melanoma cells, genistein caused only a moderate increase of intracellular Cers, which are poorly related to significant cell apoptosis. Co-administration of PDMP, a Cer glycosylation inhibitor, or SKI-II facilitated Cers accumulation and significantly enhanced genistein-induced melanoma cell apoptosis. Moreover, adding to genistein some exogenous cell-permeable short-chain Cers (C2, C4 and C6) lead to a major anti-melanoma effect by increasing cytotoxicity and apoptosis (especially C6). This mechanism could be explained by the JNK activation of and Akt inhibition.

Tiper et al. [92] showed that VEGF and ganglioside GD3 production by ovarian cancers suppress NKT- mediated anti-tumor response. The growth of cancer and the development of metastases strongly depend on the divert of the immune system response. Previous reports [93,94] showed that the ganglioside GD3 and VEGF levels in ovarian cancer ascites (OV-CAR-3 and SK-OV-3) are much higher than in ascites associated with other solid tumors. They proposed that VEGF and ganglioside GD3 synthesis pathway might be linked, working in tandem to suppress immune responses. The data proposed suggest that VEGF could modulate ganglioside GD3 expression confirming that ovarian cancer associated GD3 is responsible for suppressing CD1d-mediated NKT cell activation. This malignant overproduction of immunodepressive ganglioside could be reduced after 72 h of genistein treatment.

Phenoxodiol is a sterically modified version of genistein, with a higher bioavailability, a lower rate of metabolism and increased antitumor potency. According to Gamble et al. [95] phenoxodiol may be an effective anticancer drug, targeting the proliferation of the tumor cells and the angiogenic and inflammatory stimulation of the vasculature. These findings involve different enzymatic pathways, one of them concerning sphingolipids. It inhibited SphK which has been recently correlated with endothelial cell activation [96], angiogenesis and oncogenesis [97]. Hence, the inhibitory effect of phenoxodiol on pro-survival signals, mediated by SphK and Sph-1P, might contribute to arrest mitosis, to reduce angiogenesis and to promote apoptosis [95].

### 3.9. Luteolin

Luteolin (3′,4′,5,7-tetrahydroxyflavone) is a naturally occurring flavone, another subtype of flavonoid, found in food sources such as broccoli (*Brassica oleracea*), green chili (*Capsicum* spp.), onion leaf (*Allium unifolium*), French bean (*Phaseolus vulgaris*), carrot (*Daucus carota*), white radish (*Raphanus sativus var. longipinnatus*) and in infusion of clover blossom (*Trifolium pratense*) [67].

On a broad range of malignancies, luteolin displays different effects such as inhibition of cell proliferation, angiogenesis, metastasis, induction of apoptosis and sensitization to chemotherapy. Nevertheless, the molecular mechanisms of luteolin still remain unclear.

**Figure 5:**
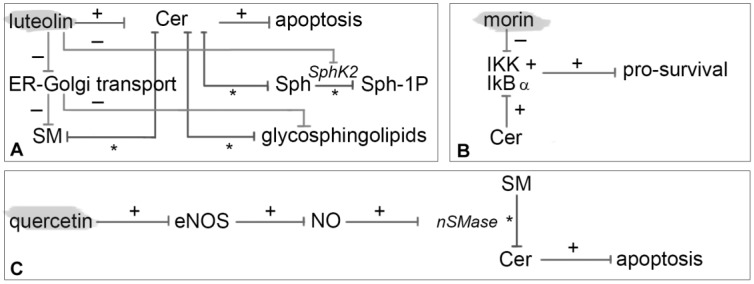
Mechanism of modulation on sphingolipids by luteolin (**A**), morin (**B**) and quercetin (**C**). It is depicted with an asterisk (*) enzymatic pathway, with plus (+) red-regulated pathway and with minus (−) down-regulation ones.

Hadi et al. [98] conducted an important study aimed to demonstrate a connection between luteolin and apoptosis in colon cancer cells. First, luteolin elevated Cer levels, followed by the apoptotic death of colon cancer cells, but not in differentiated enterocytes. Second, luteolin impaired the vesicle-mediated transport of Cer from ER to Golgi. The consequent dysregulation of sphingolipids equilibrium consisted of Cer elevation and significant reduction of both SM and glycosphingolipids. This effect may be correlated with the inhibition of AKT phosphorylation which emerges as a key mechanism affecting this vesicles route. Third, luteolin inhibited the production of Sph-1P by a SphK2 hindrance. Moreover, luteolin was proven to unbalance the sphingolipid rheostat by bending it to apoptosis in colon cancer cells (Figure 5A).

### 3.10. Morin

Morin (3,5,7,2′,4′-pentahydroxyflavone) is a flavonoid polyphenol of the class of flavonols. It is a yellow pigment that could be isolated from non-edible Osage orange (*Maclura pomifera*) and old fustic (*Maclura tinctoria*). Morin is also present in dietary infusions of white mulberry leaves (*Morus alba*), in figs (*Ficus carica*), almond (*Prunus dulcis*), guava (*Psidium guajava*) and wine [99]. Morin is a flavonol that exhibits antiproliferative, antitumor, and anti-inflammatory effects through a mechanism that is not well understood.

Manna et al. [100] proposed that morin mediates its effects by modulating NF-κB in the control of cell survival, proliferation, and tumorigenesis. NF-κB is a heterodimeric protein complex of members of the Rel protein family. NF-κB morin-mediated transcription can be promoted by a wide variety of inflammatory stimuli, including Cer (Figure 5B).

### 3.11. Quercetin

Quercetin is a naturally occurring flavonol found in high concentrations in red onions (*Allium cepa*), citrus fruits (*Citrus* spp.), apples (*Malus domestica*), red wine, and sour cherry seeds (*Prunus cerasus*) [67].

A study done by Ferrer et al. [101] showed that intravenous administration of quercetin prevented the metastatic growth of highly malignant B16 melanoma F10 cells, by enhancing NO release from the vascular endothelium through an increment of eNOS expression. The rise of NO promotes a tumor cytotoxicity and an activation of nSMase, thus increasing Cer and apoptosis.

Torres et al. [102] reported that the derivative of quercetin THDF (5,7,3′-trihydroxy-3,4′-dimethoxyflavone) inhibits cell proliferation and induces apoptosis in human leukemia cells (HL-60 and U937) by a disruption of tubulin polymerization and an activation of aSMase-dependent generation of Cer correlated with cell death (Figure 5C).

### 3.12. Resveratrol

**Figure 6:**
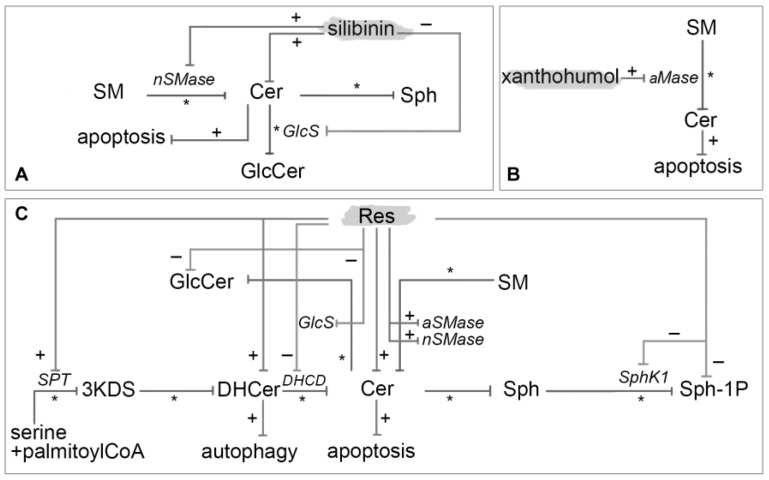
Mechanism of modulation on sphingolipids by silibinin (**A**), xanthohumol (**B**) and Res (**C**). It is depicted with an asterisk (*) enzymatic pathway, with plus (+) red-regulated pathway and with minus (−) down-regulation ones.

Res (3,5,4′-trihydroxy-trans-stilbene) is a natural stilbene found in several plants including blueberries (*Vaccinium* sect. *Cyanococcus*), mulberries (*Morus* spp.), cranberries (*Vaccinium* subgenus *Oxycoccus*), peanuts (*Arachis hypogaea*), grapes (*Vitis* spp.), rhubarb (*Rheum* spp.) and wine. It has been reported to have anti-cancer, anti-inflammatory, anti-cardiovascular disease and blood-sugar lowering properties [103,104]. It has been classified as phytoalexin for being synthesized in spermatophytes in response to injury, UV irradiation and fungal attack. It exists in both *trans*, the more frequent, and *cis* isomeric forms. In plants, Res is generally found in glycosylated forms, known as 3-*O*-β-D-glucosides, and called piceids. Other natural Res analogs contain pterostilbene and piceatannol [105]. Anticancer properties of Res are quite complex and composed of different mechanisms. It can affect the processes underlying all stages of carcinogenesis, angiogenesis and metastasis. Its activity against cancer appears to be closely associated with: mutational activation of Ras, deregulation of myc, overexpression of AP-1, amplification of cell cycle regulator cyclins D/E and Cdks 2/4, mutation of Fas and Bax, deletion of p53, disruption of DNA-damage response regulators Chk1/2 and ATM/ATR, overexpression of survival kinase AKT1, mutation of cell cycle inhibitors and translocation of anti-apoptotic Bcl-2 [106]. Here we focus on the several Res anticancer properties triggered by modulation of sphingolipid metabolism (Figure 6C).

Signorelli et al. [107] demonstrated a strict correlation between sphingolipid metabolism, Res and autophagy in gastric cancer cells (HCGC-27). Res inhibits DHCD and subsequently induces an imbalanced accumulation of DHCers *versus* Cers thus promoting autophagy rather than apoptosis.

Shin et al. [108] established that Res leads to the accumulation of endogenous Cers and significantly increases DHCers especially DHCer-C24:0 (containing lignoceric acid) in SNU-1 gastric cancer cells and HT-29 colon adenocarcinoma cells. The accumulation of DHCer with different fatty acid chain lengths (C24:0 > C16:0 > C24:1 > C22:0) was powerfully associated with Res- induced cell cytotoxicity although the inhibition of DHCD was not found to be a critical mechanism. The effect of Res was drastically increased by dimethylsphingosine (a non-specific SphK inhibitor) and retinamide (4-HPR, a non-specific DHCD inhibitor) but not by GT-11 (a specific DHCD inhibitor). The Res cytotoxic effect is cell-specific: SNU-1 and HT-29 are highly sensitive in contrast with SNU-668.

According to Lin et al. [109], Res and Cer could be used in sequence or in combination for chemoprevention and cancer treatment due to their similarities in transduction pathways to induce apoptosis in human ovarian cancer OVCAR-3 cells. Cer and Res uses an endocytic- and activated ERK1/2 dependent pathway to induces apoptosis in human ovarian cancer cells. Additionally, exposure to these compounds induces expression and nuclear accumulation of COX-2 without affecting COX-1, Ser-15 phosphorylation of p53 and accumulation of BcL-xS. By contrast, only Cer utilizes both p38 kinase-dependent pathway and ERK 1/2-dependent pathway whereas Res only the latter one. However, the relationship of COX-2 protein on cancer is not easy to establish: some studies reported an expression of COX-2 in cells associated with tumor cell growth, metastasis, enhanced cellular adhesion and inhibition of apoptosis [110] whereas others suggested a pro-apoptotic activity [111].

Lim et al. [112] showed that Res and its dimers (ampelopsin A and balanocarpol) could perturb SphK 1-mediated signaling in MCF-7 breast cancer cells. Ampelopsin A and balanocarpol are dimers of Res formed by the fusion of *cis*- and *trans*-isomers and they could be extracted and isolated from plants in the Dipterocarpaceae family. In this family *Hopea dryobalanoides* and *Hopea odorata* supply a very limited food products. In this study, Res was found to be a competitive inhibitor of SphK1 and balanocarpol is about twice as potent as Res on kinase inhibition because of its binding to two catalytic sites simultaneously. The mechanism of down-regulation of SphK1 expression might involve changes in its protein turnover by ubiquitin-proteasomal or modification in lysosomal-cathepsin B proteolysis or alterations in gene promoter activity.

In agreement with Lim et al. [112], Tiang et al. [113] proposed Res to be an apoptotic agent in the myelogenous leukemia cell line K562 by modulation of SphK1 and translocation of the enzyme from the membrane to the cytosol. The kinase activity is clearly repressed granting a restoration of sphingolipid balance. Sph-1P level decreases whereas Cer level increases. 

Cakir et al. [114] showed that Res induces apoptosis through a concurrent increase of *de novo* Cer and decrease of anti-apoptotic Sph-1P and GlcCer. Not only, targeting Cer metabolism increased chemosensitivity to Res in acute myeloid leukemia cells.

Kartal’s study [115] was also focused on the relationship between the sphingolipid pathway, Res and human K562 chronic myeloid leukemia cells. A synergistic anti-proliferative effect was observed with Res in combination with: (1) Cer-C8, a cell-permeable analog of natural Cer inducing *de novo* generation; (2) PDMP, an inhibitor of GlcS; and (3) PF-543, a SphK1 inhibitor. Moreover, they showed that Res triggers apoptosis through raising expression of longevity assurance genes (LASS2, LASS4, LASS5, LASS6) correlated with down-regulation of GlcS and SphK 1.

Chow et al. [116] reported an abnormal accumulation of Cer *via* activation of SPT resulting in an ER dilation/expansion and thus ER stress. ER stress is, indeed, firmly associated with cell apoptosis by mechanisms involving direct activation of ER-associate caspases (3, 9 and 12) and CHOP, a common downstream pro-apoptotic molecule of unfolded protein response.

Wang et al. [117] described two divergent mechanisms of Res in melanoma B16 cells. They showed an inhibition of B16 cell growth *via* induction of mitochondrial apoptosis and contemporary inducing protective autophagy through Cer accumulation and AKT/mTOR pathway inhibition. Interruption of the autophagy program leads to an improvement of the efficacy of Res cytotoxicity and apoptosis. It was the first study revealing that Res-induced accumulation of Cer conferred protection of B16 cells against apoptosis inducing protective autophagy.

Another mechanism was proposed according to Mizutani et al. [118]. Inhibition in K562 (a human leukemia cell line) and HTC116 (a human colon cancer cell line) by Res was correlated to up-regulation of Cer and aSMase expression and down-regulation of Sph-1P. This study suggested a possible relationship between Res-induced cell growth inhibition and the sphingolipid metabolism modulation.

As previously mentioned, catechin and Res synergically inhibit SphK1 activity, *via* a novel ERK/PLD-dependent mechanism in prostate cancer cells (C4-2B hormone-responsive and PC-3 hormone- refractory) acting as a possible anti-cancer effector [40].

According to Scarlatti et al. [119] activation of the *de novo* Cer synthesis by Res is the mechanism underlying its growth inhibitory effect on the metastatic, drug-resistant and highly invasive breast cancer cell line MDA-MB-231. This accumulation derives from both *de novo* Cer synthesis and SM hydrolysis by activation respectively of SPT and nSMase.

Another work by Scarlatti et al. [120] presented that pretreatment with Res enhances tumor cell killing and inhibits the clonogenic survival in resistant irradiated-DU145 prostate cancer cells, synergistically affecting the cellular response to ionizing radiation. This event was mediated by an increase in cellular *de novo* Cer levels.

Dolfini et al. [121] demonstrated that targeting Cer signaling with Res might offer a potential strategy to prevent the growth of hormone-independent breast cancer. Res exerts a severe inhibitory effect on the growth of MDA-MB-231 both *in vitro* and *in vivo*. It affects the aggregation properties of MDA-MB-231 cells into multicellular tumor spheroids in association with induction of *de novo* synthesis of Cer.

Minutolo et al. [122] showed that a synthesized derivative of Res [5-(6-hydroxynaphthalen-2-yl)benzene-1,3-diol] is more effective in triggering apoptosis, coupled with the induction of endogenous Cer in human cancer cells MDA-MB-231. Since the Res biological activity in cancer cells is limited by its photosensitivity and metabolic instability, the authors replaced the 3,5-hydroxy groups with more stable methoxy groups, thus obtaining a compound with increased anti-proliferative activity. Moreover, the stabilization of the stilbene double bond of Res by a naphthalene ring increases the molecular rigidity. This dramatically improves the biological activity *via* Cer-mediated pro-apoptotic mechanism coupled to cleavage of PARP.

### 3.13. Silibinin

Silibinin is the most active and major component (60–70%) of silymarin, a standardized extract from the seeds of the milk thistle seeds (*Silybum marianum*). Other flavonolignans consist in silibinin, isosilibinin, silychristin, isosilychristin and silydianin. Silibinin is a mixture of two diastereomers, silybin A and silybin B, in approximately equimolar ratio [123].

It has been used in the prevention and treatment of viral hepatitis, cirrhosis caused by alcohol abuse and liver damage caused by medications or industrial toxins, in traditional and modern medicine. Silibinin effects are due to free radical trapping, prevention of lipid peroxidation, an increment of proapoptotic protein (Bax, p53), a decrement of anti-apoptotic proteins (Bcl-2 and Bcl-xL) and anti-cancer activity.

Boojara et al. [124] investigated the effects of four silibinin derivatives that is silybin A, silybin B, 3-*O*-galloyl-silybin A and 3-*O*-galloyl-silybin B on cell viability, caspase assessment, total Cer levels and Cer-metabolizing enzyme in Hep G2 hepatocarcinoma cell line. Exposure to silibinin isomers and gallate derivatives in human liver carcinoma cells resulted in increased Cer levels. Gallate derivatives had a stronger ability in Cer elevation in comparison with silybin A and B. The activity of aCDase, the enzyme involved in the catabolism of Cer to Sph, was markedly inhibited by silybin B, 3-*O*-galloyl-silybin A and 3-*O*-galloyl-silybin B. The activity of nSMase was increased by treatment with silybin A, silybin B and 3-*O*-galloyl-silybin A whereas the activity of GlcS was inhibited by silibin A, silibin B and 3-*O*-galloyl-silybin B (Figure 6A).

### 3.14. Xanthohumol

Xanthohumol (3′-[3,3-dimethyl allyl]-2′,4′,4-trihydroxy-6′-methoxychalcone) is the principal prenylated chalcone of the female inflorescences of the hop plant (*Humulus lupulus*). It is the main ingredient of beer and together with prenylflavonoids it is used to add bitterness and flavor. The naturally occurring chalcones are heat-degraded during the brewing process therefore relatively high levels are due to a second addition of hops to the boiling wort.

Xanthohumol has been shown to elicit anti-inflammatory, antiangiogenic, anticancer, antibacterial, antifungal, antimalarial and antiviral effects. It favorably influences also sleep disorders and menopausal symptoms in women, acting as estrogen by its metabolites isoxanthohumol and 8-prenylnaringenin. According to Xuan et al. [125] xanthohumol stimulates aSMase in dendritic cells, derived from mouse bone marrow, leading to Cer formation and caspase activation. The sequence of events postulated was: (1) translocation of aSMase onto cell surface; (2) formation of Cer; (3) autocatalysis of caspase 8; (4) activation of caspase 3; and (5) DNA fragmentation and proteolysis of intracellular proteins (Figure 6B).

## 4. Conclusions

Cancer treatment and cancer prevention are a constant challenge for clinicians and the whole scientific community. Nutrients on their own appear to offer a good strategy in prevention more than in cancer therapy. However, chemotherapy has gradually transitioned from monotherapy to multidrug therapy. It is believed that a combination of classical chemotherapy with nutrients and especially with polyphenols dietary sources may improve efficacy and decreases negative side effects of the antineoplastic drug. In this multifaceted scenario, sphingolipids play a pivotal role as bioactive molecules, controlling several aspects of cancer from cell growth and proliferation to anti-cancer therapeutics. Further research on the crosstalk between polyphenols and sphingolipids could lead to better understand their reciprocal roles and to develop new therapeutic strategies against cancer.

## Figures and Tables

**Table 1:** Polyphenols classes and examples of more relevant compounds.

**Flavonoid Polyphenols**	
Flavones	apigenin, chrysin, diosmin, luteolin, baicalein
Isoflavones	daidzein, daidzin, genistein
Flavanones	hesperetin, narigenin
Flavonols	kaempferol, quercetin, rutine, myricetin, morin
Anthocyanidins	cyanidin, dephinidin, malvidin, pelargonidin, peonidin
Chalcones	butein, curcumin, xanthohumol
Flavanols	catechins, tannins
**Non-Flavonoid Polyphenols**	
Benzoic acids	vanillic acid, gallic acid, syringic acid
Cinnamic acids	caffeic acid, chlorogenic acid, CAPE, tannic acid
Stilbenes	resveratrol, piceatannol, isorhapontigenin, oxyresveratrol

**Table 2:** Roles of sphingolipids in cancer.

Sphingolipids	Biological Target	Effect in Cancer	References
Cer	PKC, I2PP2A, cathepsin D, caspases, telomerase	Apoptosis, growth arrest, senescence	[23,26,27,28,29,30,31,34,35,36,37]
Cer-1P	cPLA_2_	Release of arachidonic acid and activation of inflammatory cascade	[31,33,37]
DAG (from SM)	PKC	Cellular proliferation	[38,39]
Sph-1P	NFKB, COX-2, ERK	Malignant transformation, anti-apoptosis, angiogenesis, survival, metastatization	[31,32,33,40]

## Abbreviations

3-KDS	3-keto dihydrosphingosine
4-HPR	retinamide
aCDase	acid ceramidase
AIF	apoptosis inducing factor
AKT	protein kinase B
AP-1	activator protein 1
aSMase	lysosomal acidic sphingomyelinase
ATF6	activating transcription factor-6
ATM	ataxia-telangiectasia mutated kinase
ATR	serine/threonine-protein kinase ATR or ataxia telangiectasia and Rad3-related protein
BAX	apoptosis regulator BAX
BAX	apoptosis regulator BAX
Bcl-2	B-cell lymphoma 2
Bcl-xL	B-cell lymphoma-extra large
Bcl-xS	B-cell lymphoma-xS
BCR/ABL	Philadelphia chromosome
c-FOS	cellular DNA-binding proteins encoded by the c-fos genes
CAPE	caffeic acid phenethyl ester
CDase	ceramidase
Cdc25C	gene for M-phase inducer phosphatase 3
Cdk1	cyclin-dependent kinase 1
Cer	ceramide
CERT	ceramide transfer protein
Cer-1P	ceramide-1-phosphate
CerK	ceramide kinase
CerS	ceramide synthases
CERT	ceramide transfer protein
cGMP	cyclic guanosine monophosphate
Chk1/2	checkpoint kinase ½
CHOP	C/EBP homology protein
CHOP	transcription factor CCAAT-enhancer-binding protein homologous protein
COX	cyclooxygenase
COX-2	cyclooxygenase 2
cPLA2	cytosolic phospholipases A2
CREB	cAMP response element-binding protein
DHCD	dihydroceramide desaturase
DHCer	dihydroceramides
DHSph	dihydrosphingosine or sphinganine
EGCG	epigallocatechin-3-gallate
EGF	epithelial growth factor
ELISA	enzyme-linked immunosorbent assay
ENOX	Ecto-NOX disulfide-thiol exchanger
ENSA	electrophoretic mobility shift assay
ER	estrogen receptor
ER	endoplasmic reticulum
ERK	extracellular signal regulated kinase
FACS	fluorescence activated cell sorted
Fas	first apoptosis signal receptor
FITC	fluorescein isothiocyanate
GalCer	galactosylceramide
GC/MS	gas chromatography tandem mass spectrometry
GD3	ganglioside GD3 or disialosyllactosylceramide
GlcCer	glucosylceramide
GlcS	glucosylceramide synthase
GPR30	G protein-coupled receptor for estrogen
GSK-3	glycogen synthase kinase 3
GT-11	*N*-[(1*R*,2*S*)-2-hydroxy-1-hydroxymethyl-2-(2-tridecyl-1-cyclopropenyl)ethyl]octanamide
GW4869	*N*,*N*′-Bis[4-(4,5-dihydro-1*H*-imidazol-2-yl)phenyl]-3,3′-p-phenylene-bis-acrylamide
HIF-1α	hypoxia-inducible factor-1α
HNSCC	head and neck squamous cell carcimona
HPLC	high performance liquid chromatography
HPTLC	high performance thin layer chromatography
I2PP2A	protein phosphatase 2A inhibitor 2
IKK	IκBα kinase
IκBα	inhibitory subunit of NF-κB
JAK	janus kinase
JNK	c-Jun N-terminal kinase
KDSR	3-ketodihydrosphingosine reductase
LacCer	lactosylcercamide
LASS	longevity assurance genes
Lc3	GlcNAcβ1-3Galβ1-4Glcβ-Cer
LPS	lipopolysaccharides
MAPK	mitogen activated protein kinase
MPM-2	mitotic protein monoclonal 2
mTOR	mammalian target of rapamycin
MTT	3-(4,5-dimethylthiazol-2-yl)-2,5-diphenyltetrazolium bromide
NADH	reduced form of Nicotinamide adenine dinucleotide
NF-κB	nuclear factor kappa-light-chain-enhancer of activated B cells
NGF	nerve growth factor
NGFR	nerve growth factor receptor
nSMase	neutral sphingomyelinase Mg-dependent
P-gp	permeability glycoprotein
p38 MAPK	p38 mitogen activated protein kinase
p53	tumor protein p53
p75NTR	low-affinity nerve growth factor receptor or p75 neurotrophin receptor
PA	phosphatidic acid
Par-4	prostate apoptosis response 4
PARP	poly (ADP-ribose) polymerase
PDMP	dl-threo-1-phenyl-2-decanoylamino-3-morpholino-1-propanol
PI3K	phosphatidylinositide 3-kinases
PKC/PKA	protein kinase C/protein kinase A
PLC	phospholipase C
PLD	phospholipase D
PPAR	peroxisome proliferator-activated receptors
PPE	polyphenon E
Res	resveratrol
ROS	reactive oxygen species
S1PR1-5	sphingosine-1-phosphate receptors 1–5
SEAP	secreted embryonic alkaline phosphatase
SKI-II	2-(p-Hydroxyanilino)-4-(p-chlorophenyl)thiazole
SM	sphingomyelin
SMase	sphingomyelinase
SMS1/2	sphingomyelin synthase 1/2
Sph	sphingosine
Sph-1P	sphingosine-1-phosphate
SphK1/2	sphingosine kinase 1/2
SPL	sphingosine-1-phosphate lyase
SPPase1/2	sphingosine-1-phosphate phosphatases 1/2
SPT	serinepalmitoyl transferase
STAT	signal transducer and activator of transcription protein
THDF	5,7,3′-trihydroxy-3,4′-dimethoxyflavone
THDF	5,7,3′-trihydroxy-3,4′-dimethoxyflavone
TLC	thin layer chromatography
TLC	thin layer chromatography
TNF	tumor necrosis factor alpha
VDAC	voltage-dependent anion-selective channel
VEGF	vascular endothelial growth factor
XM462	Octanoic acid (1*S*,2*S*)-(2-hydroxy-1-hydroxymethyl-3-tridecylsulfanyl-propyl)-amide
